# The Law of the Similars the *Only* Law of Cure

**Published:** 1887-12

**Authors:** Ad. Lippe

**Affiliations:** Philadelphia


### THE LAW OF THE SIMILARS THE *ONLY* LAW OF CURE IN THE TREATMENT OF THE SICK.

AD. LIPPE, M. D., PHILADELPHIA.

As the homœopathic healing art is based upon the law of the similars, there can never be in existence exceptional cases which require in their treatment a deviation from this law of nature. If the law is the only law for the *cure* of the sick, there cannot be other laws also applicable for the *treatment* of the sick.

The very admission that exceptional cases not only permit but absolutely require the adoption of other ways of treating the sick opens wide the door for the practice of eclecticism, *vide The North American Journal of Homœopathy*, August, 1887, especially page 492. Adjuvant treatment indeed! Is not such a thing an absolute refutation of the claim to universal application of the law of the similars in the treatment of the sick? According to that authority (said journal is the organ of the New York Homœopathic College), Homœopathy does not possess means (under the law of the similars) to cure the violent colics caused by gallstones or kidney calculus. The means advocated in the aforesaid article are Morphia administered hypodermically for the relief of what, the disease? no, the mechanical pressure. The statement there made by the organ of a homœopathic college is erroneous from first to last. Homœopathy does *cure* gallstones and kidney calculus under the homœopathic law if the similar remedy is *properly* administered. Morphia hypodermically injected may relieve (palliate) the pain, but the relief is a deception, the sick is not cured, and before long becomes convinced of it by renewed attacks of increased severity and frequency. The old veterans who gave Homœopathy a status *cured* and continued to cure all such cases, and is it now to be declared "a lost art" to cure without adjuvant treatment!

Some *opinions* have lately been advanced that Homœopathy is not the only way to treat the sick, while it is acknowledged that the law of the similars is the only law of cure. The opinion is expressed that it is rare that a remedy homœopathic to the symptoms fails to relieve even in an incurable case, and if it so happens, whether from the ignorance of the physician *or* the peculiarity of the case, one's *duty* is perfectly clear and must be followed—the duty is to give Opium or Morphia. According to this *opinion*, the more ignorant the physician the oftener it will be his duty to give Opium, and the less ignorant the physician the less often it will be his duty to give it, and if not ignorant the duty to give it will *never* arise. Hahnemann, the founder of our healing art, proved Opium, and it would benefit the advocates of Opium to read the exhaustive preface in the first volume of Hahnemann's *Reine Arzneimittel Lehre.* The most frequently indicated remedies under the unerring laws of the similars to relieve the agonies of the incurably sick are Arsenic, Rhus tox., Lachesis, and Tarantula. These may, in some peculiar cases, not be indicated, but the conscientious healer not ignorant of our materia medica will find in even peculiar cases the proper remedy. If the law of the similars has correctly guided him in other cases there can be no plausible exception to the applicability of it.

Another opinion is expressed that the cause of the disease must be forcibly removed and then the patient be homœopathically treated, thereby preventing a recurrence of the malady. These causes include substances visible under the microscope. If, so the opinion runs, the patient suffering from a contagious or zymotic disease is in a very feeble condition and not able to resist it, we must suppress for a time the violence of the zymosis. Quinine for intermittent fever, suppresses for a time the violence of the zymosis, and then the cure of the patient is made homœopathically. That sounds plausible but it does not work. The intermittent fever suppressed for a time by powerful doses of Quinine *must* return again as soon as homœopathic treatment begins, if it does not the Quinine and malarial cachexia will set in to the final detriment of the sick. Again, the *opinion* is given that every healer should step out and down who will not cure in twenty-four or forty-eight hours a fresh case of gonorrhœa by injecting a watery solution of the five thousandth of a grain of Corrosive Mercury! There are no specifics for specific diseases that any homœopath knows of, and it will not do to profess to be a homœopath and advocate such treatment; this is a local application for an erroneously supposed local disease. Local applications in nonsurgical cases are never salutary. Ulcers may be dried up by local treatment, but the patient does not recover.

If the law of the similars is the only law of cure, and if the homœopathic school accepts the teachings of Hahnemann as recorded in his *Organon of the Healing Art*, opinions in contravention of his advice for applying the law of the similars for the cure of the sick will not mislead any true healer. Modern medical men profess to have found by the aid of the microscope the *prima causa morbi*, and we are asked to remove the cause of the disease. What has been found by the microscope is the product of the disease. By removing these products we do not cure the disease, and what Hahnemann says on that point in paragraph six and foot-notes of the *Organon* is as true to-day as it was then—just as true as anything he ever said. In the seventh paragraph of the *Organon* he dwells on the *causa occasionalis*, and every sensible physician of any school will always first remove the evidently occasioning and sustaining cause of the sickness. Many of the causes are mentioned in the foot-note to that paragraph.

The frequently recurring expression of different opinions how best to practice Homœopathy can easily be settled by referring to the teachings of Hahnemann. *Opinions* not in harmony with his teachings or *opinions* not sustained by ordinary logic are harmless amusements to those who express them. One amused himself by attempting to show that it was in accordance with the teachings of Hahnemann that morbific products *if* highly potentized would cure the disease itself. What of it? A splutter, and now he is as silent as a clam. Before long a large majority of healers will come to the conclusion that there is and can be only one law of cure in the treatment of the sick—the law of the similars—and only one teacher who taught how to apply that law. That teacher, the founder of the homœopathic healing art, Hahnemann, will not be excelled by any modern prophet who tramples on the *Organon* and holds as an emblem of his adherence to Homœopathy in one hand the microscope, in the other the Quinine bottle, and in his buttonhole a hypodermic syringe. The recognition seekers will be *recognized*.

